# Anatomical Measurement as a Reference for Functional Endoscopic Sinus Surgery Based on CT Scans and Dissections

**DOI:** 10.1055/s-0043-1777447

**Published:** 2024-07-05

**Authors:** Andressa Vinha Zanuncio, Flávia Gontijo Amaral, Marcell de Barros Duarte Pereira, Flávio Barbosa Nunes, Roberto Eustáquio Santos Guimarães

**Affiliations:** 1 Department of Medicine, Universidade Federal de São João del-Rei, Campus Centro Oeste, Divinópolis, MG, Brazil; 2 Department of Radiology, Universidade Federal de São João del-Rei, Divinópolis, MG, Brazil; 3 Department of Medicine, Universidade Federal de Minas Gerais, Belo Horizonte, MG, Brazil

**Keywords:** endoscopic surgery, FESS, skull base, paranasal CT scans, paranasal sinuses

## Abstract

**Introduction** Diseases of the paranasal sinuses, nasal cavities, and those related to the skull base can be treated with nasal endoscopic surgery. Anatomical references are essential to safely perform these surgeries.

**Objective** To measure and compare the distance from the posterior wall of the maxillary sinus to the anterior skull base in cadavers and on computed tomography (CT) scans to determine a measurement as an anatomical reference in imaging exams for sinus and anterior skull base surgery.

**Methods** In dissections and CT scans, we took measurements from the most upper and medial point of the posterior wall of the maxillary sinus (point A) to the point where the skull base deflects and the anterior sphenoid wall is formed (Δ 90°; point B), in the right and left nasal cavities. We used 51 cadavers aged ≥ 18 years in the present research.

**Results** The measurements obtained from CT scans and dissections were greater than 1.5 cm in all cadavers, and they were positively correlated. The 1-cm increase in the AB-tomography measurement corresponded to the 1.08-cm increase to the right and 1.07-cm to the left in the AB-dissection measurement.

**Conclusion** The CT measurements may be considered a reliable tool to promote safe and effective access to the paranasal sinuses, matching the distance that should be dissected until the anterior base of the skull.

## Introduction

Advances in paranasal sinus surgery have enabled the use of endoscopic techniques to treat diseases of the sinuses and anterior skull base. The use of the rigid endoscope, with various angles, enables the visualization of the paranasal sinuses from the ostia to their internal part, as well as the assessment of pathologies without the need for external access and with lower complication rates. 1

The surgeon needs to know the anatomy of the paranasal sinuses and the locations used as anatomical references for access. Anatomical variations are common and should be expected during surgery. The absence of anatomical references, such as the uncinate process, is greater in repeat surgeries. 2 3

Computed tomography (CT) scans of the paranasal sinuses and videonasolaryngoscopy are mandatory for diagnosis and surgical planning. The exam is used during the surgical procedure to guide dissection through the identification of anatomical structures and their variations. 4 The proper identification of noble structures, such as the optic nerve, is important due to individual variations in CT scans 5 6 and in cadaver dissections. 7

An appropriate surgical approach to the paranasal sinuses may increase surgical success, leading to lower morbidity and higher disease resolution. However, synechiae, anosmia, liquoric fistulas, 8 as well as lesions in noble structures, may cause irreversible damage. 9 10

Quality of life assessments and clinical follow-up of patients with chronic sinusopathy submitted to surgical treatment are considered the appropriate interventions to ensure that patients remain symptom-free for longer periods. 11

Knowing the anatomical references and fixed measurements with almost no variation in terms of gender, race, age, weight, and height might provide surgeons more reliability, minimizing complications and optimizing interventions in the nasal sinuses. 12

The hypothesis of a regular distance, greater than 1.5 cm, between the posterior wall of the maxillary sinus and the skull base has been presented in a research with 120 nasal cavities of cadavers. 12 The purpose of the present research was to measure and compare the distance from the posterior wall of the maxillary sinus to the anterior skull base in cadavers and on CT scans to determine a measurement as an anatomical reference in imaging exams to assist the surgeon in endoscopic nasal surgeries.

## Methods

The present study was approved by two Ethics in Research Committees on Plataforma Brasil.

The right and left nasal cavities of 57 cadavers aged ≥ 18 years were dissected and submitted to CT scans. Six were excluded: three because the measurements had been taken without the camera, two due to maxillofacial complex fracture and one due to poor quality of the CT scan. Finally, 51 cadavers were included in the study, totaling 102 nasal cavities. The cadavers were included according to the occurrence of deaths, regardless of gender, age, height, race, or cause of death.

The paranasal sinus CT scans in the axial plane were performed prior to dissection using a multislice16-channel CT scanner (GE Healthcare, Chicago, IL, United States). The two anatomical reference points were visualized in the same image with multiplanar reformatting (MPR) in the 0.65-mm thick sagittal plane.

**Fig. 1:**
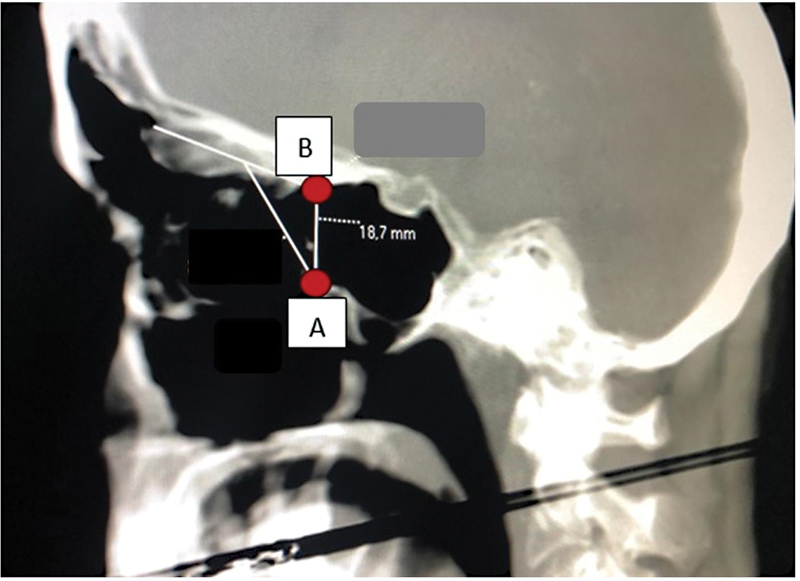
Sagittal plane (tomography): points A (Δ 90°) and B (maxillary sinus posterior wall) - distance.

Measurements were taken from the most upper and medial point of the posterior wall of the maxillary sinus (point A) to the point where the skull base deflects and the anterior sphenoid wall is formed (Δ 90°; point B) ( Fig. 1 ). Both sides were measured. The measurements were performed by two radiologists, and the level of agreement between them was analyzed to determine how reapplicable the measurement might be.

After the CT scans, the cadavers were sent to the hospital's morgue, where the structure for dissection was assembled with surgical instruments, video monitor, light source, vacuum cleaner, and camera.

**Fig. 2:**
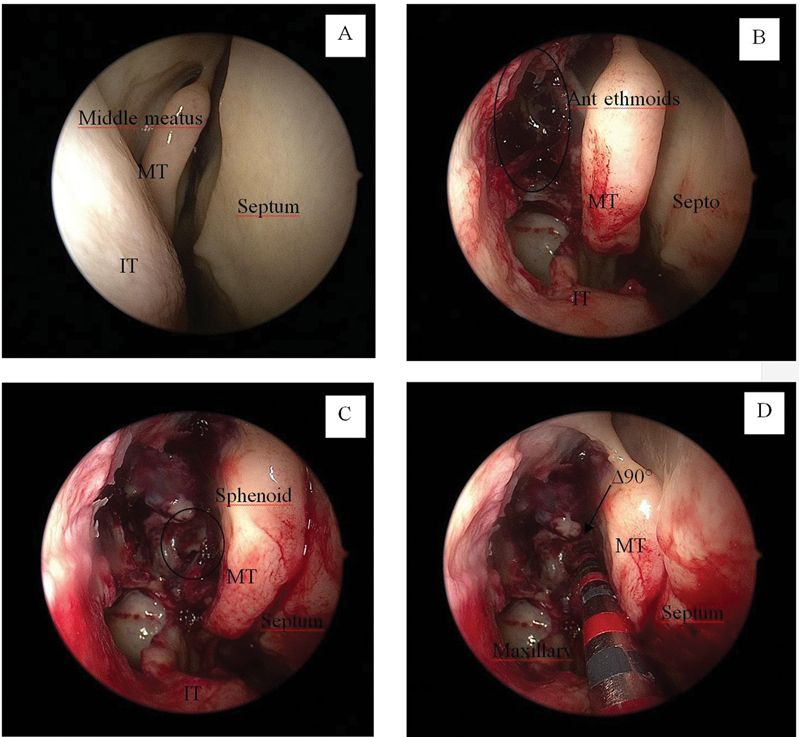
Right nasal cavity. ( **A** ) Beginning of dissection; ( **B** ) Maxillary sinus and anterior ethmoidal complex; ( **C** ) Sphenoid sinus, ( **D** ) Δ 90°; a scalpel with a ruler was used for the measurements. Abbreviations: MT, middle turbinate; IT: inferior turbinate.

**Fig. 3:**
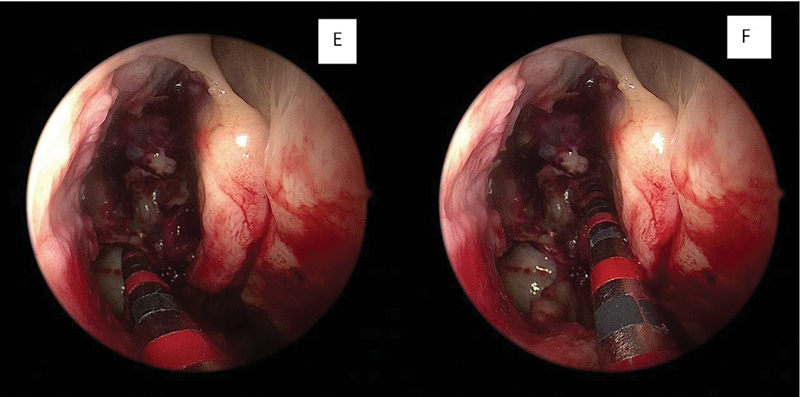
AB-dissection measurements. E: posterior wall of the maxillary sinus – point A / F: Δ90° – point B.

The dissections of the right and left nasal cavities were made by endoscopic maxillary, ethmoid, and sphenoid sinusotomy to identify points A and B ( Fig. 2 ). All dissections and measurements were performed by the same researcher, with a scalpel with a ruler inserted into the nasal cavity. Initially, the scalpel is supported at point A and the measurement is made; then, it supported at point B, and a new measurement is made. The difference between both is the distance between points A and B (AB measurement) ( Fig. 3 ).

The dissections and measurements were photographed and recorded in the research files. No biological material was removed from the cadavers, which underwent no changes in physiognomy.

The parameters in the present research were the measurements obtained from the cadavers, CT scans, and the relationship between them.

The first analysis, called “AB-tomography,” was performed on CT scans through the AB measurement ( Fig. 1 ), and the number of measurements > 1.5 cm was recorded.

In the second analysis, called “AB-dissection,” the AB measurement was made and values > 1.5 cm were analyzed ( Fig. 3 ).

The measurements from both analyses were compared to determine a measurement as an anatomical reference with lower variation to provide reliability while treating diseases of the paranasal sinuses and the anterior skull base.

## Results

The statistical analyzes were performed using the R (R Foundation for Statistical Computing, Vienna, Austria) software, version 3.2.5, and the PASW Statistics for Windows (SPSS Inc., Chicago, IL, United States) software, version 18.0, considering a significance level of 5%.

### Descriptive Analysis

The results of the descriptive analysis were expressed ad frequencies and percentages for the qualitative variables and as measures of central tendency (mean and median) and dispersion (standard deviation, SD) for the quantitative variables.

### Comparisons

#### Agreement between Radiologists

The agreement between the two radiologists for the AB-tomography on both sides was assessed through the intraclass correlation coefficient (ICC), whose values were interpreted using the same scale as that of the study by Menz et al. 13 ( Table 1 ).

**Table 1:** Evaluation of the intraclass correlation coefficient (ICC) of the measurements made by the radiologists

ICC	Rating
≥ 0.75	Excellent
0.40 to 0.75	Moderate
< 0.40	Poor

**Note:** Adapted from Menz et al. 13

The AB-tomography measurements for both sides were compared using the paired *t* -test, since the assumption of normality assessed through the Kolmogorov-Smirnov test was not violated.

The AB-dissection and the AB-tomography measurements for both sides made by radiologist 1 were compared using the Pearson correlation analysis, Bland–Altman method, and linear regression analysis to assess the correlation, agreement, and to quantify the association between them, since the assumption of normality assessed by the Kolmogorov-Smirnov test was not violated.

## Results of the Descriptive Analysis

The age range of the 51 cadaver specimens was as follows: 11 to 20 years –1 (2.0%); 21 to 30 years – 2 (4.0%); 31 to 40 years – 1 (2.0%); 41 to 50 years – 5 (9.8%); 51 to 60 years – 11 (21.5%); 61 to 70 years – 14 (27.4%); 71 to 80 years – 9 (17.6%); and 81 to 90 years – 8 (15.7%). Regarding gender, 20 (39.2%) specimens were female and 31 (60.8%) were male, with 44 (86.3%) deaths due to natural causes and 7 (13.7%) due to violent causes.

In all cases, all of the AB-dissection and AB-tomography measurements for both sides, were greater than 1.5 cm ( Table 2 ); their description in quantitative form and stratified by side can be seen in Table 3 ).

**Table 2:** Description of the AB-dissection and AB-tomography measurements performed by two radiologists on both sides

Aspects analyzed	Frequency (%)	
	< 1.5 cm	≥ 1.5 cm
**Right side**		
AB-dissection	0 (0.0%)	51 (100.0%)
AB-tomography: radiologist 1	0 (0.0%)	51 (100.0%)
AB-tomography: radiologist 2	0 (0.0%)	51 (100.0%)
**Left side**		
AB-dissection	0 (0.0%)	51 (100.0%)
AB-tomography: radiologist 1	0 (0.0%)	51 (100.0%)
AB-tomography: radiologist 2	0 (0.0%)	51 (100.0%)

**Table 3:** Quantitative description of the AB-dissection and AB-radiologist (1 and 2)measurements performed by the two radiologists on both sides

Item	*N*	Mean	Standard deviation	Minimum	1st Q	3rd Q	Maximum
Right side							
AB-dissection	51	2.1	0.2	1.6	2.0	2.0	2.8
AB-radiologist 1	51	1.9	0.2	1.6	1.8	2.0	2.6
AB-radiologist 2	51	1.9	0.2	1.6	1.8	2.0	2.6
Left side							
AB-dissection	51	2.0	0.2	1.8	2.0	2.0	2.5
AB-radiologist 1	51	1.9	0.2	1.6	1.8	2.0	2.5
AB-radiologist 2	51	1.9	0.2	1.6	1.8	2.0	2.5

**Abbreviations:** 1st Q, 1st quartile; 3rd Q, 3rd quartile.

On the right side, the AB-dissection measurements were of 2.1 cm on average, with an SD of 0.2 cm and a mean of 2.1 cm, while the AB-tomography measurements were of 1.9 cm with an SD of 0.2 cm and a mean of 1.9 cm made by radiologist 1; the same measurements for both analyses made by radiologist 2 were of 1.9 cm, with an SD of 0.2, and a mean of 1.9 cm (Table 3).

On the left side, the AB-dissection measurements made by both radiologists were of 2.0 cm on average, with an SD of 0.2 cm and a mean of 2.0 cm, while those for the AB-tomography were of 1.9 cm, with an SD of 0.2 cm and a mean of 1.9 cm ( Table 3 ).

### Comparisons

#### Agreement between Radiologists

The ICC with its respective 95% confidence intervals (95%CIs) and the agreement between AB-tomography measurements are presented ( Table 4 ). Values of *p* < 0.05 indicated results with statistical significance, with agreement between the two radiologists, regardless of the side evaluated, and classified as excellent (ICC: right side – 0.92; and left side – 0.90). The AB-tomography measurements performed by the two radiologists were similar ( *p* -values > 0.05) ( Table 5 ).

**Table 4:** Agreement between the AB-tomography measurements made by the radiologists on both sides

Side	ICC	95%CI	Classification	*p-* value
Right	0.92	0.8–0.96	Excelent	< 0.001
Left	0.90	0.83–0.94	Excelent	< 0.001

**Abbreviations:** 95%CI, 95% confidence interval; ICC, intraclass correlation coefficient.

**Table 5:** Comparisons of AB-tomography measurements made by radiologists on both sides

Side	AB-measure								*p* -value
	Radiologist 1				Radiologist 2				
	*n*	Mean	Standard deviation	Median	*n*	Mean	Standard deviation	Median	
Right	51	1.9	0.2	1.8	51	1.9	0.2	1.9	0.547
Left	51	1.9	0.2	1.9	51	1.9	0.2	1.9	0.176

Note: Paired t-test.

#### Correlation

The correlations between the AB-dissection and AB-tomography measurements made by radiologist 1 on both sides are shown in Table 6 . The Pearson correlation coefficient on the right and left side were of 0.88 (95%CI: 0.80–0.93) and 0.76 (95%CI: 0.62–0.86) respectively, both with a *p* -value < 0.001. These results indicate a positive and strong correlation between the measurements and that an increase or decrease in the AB-dissection measurements is followed by an increase or decrease in the AB-tomography measurements made by radiologist 1.

**Table 6:** Correlation between AB-dissection and AB-tomography measurements performed by radiologist 1 nasal cavities

Nasal cavity	Coefficient ^a^	95% confidence interval	*p* -value
Right	0.88	0.80–0.93	< 0.001
Left	0.76	0.62–0.86	< 0.001

Note: ^a^ Pearson correlation coefficient.

#### Agreement

The agreement between the AB-dissection and AB-tomography measurements made by radiologist 1 was based on Bland–Altman plot analysis. Initially, the differences between the AB-dissection and AB-tomography measures and the means between the measures are calculated. Then, the mean and SD of these differences are calculated. With these results, the 95%CI for the mean of the differences for each evaluated side is obtained ( Table 7 ).

**Table 7:** Mean and standard deviation of the differences in measurements and confidence interval for the mean of the differences

Nasal cavity	Mean of the differences	Standard deviation of differences	95% confidence interval
Right	0.15	0.11	− 0.08–0.37
Left	0.14	0.12	− 0.09–0.36

**Fig. 4:**
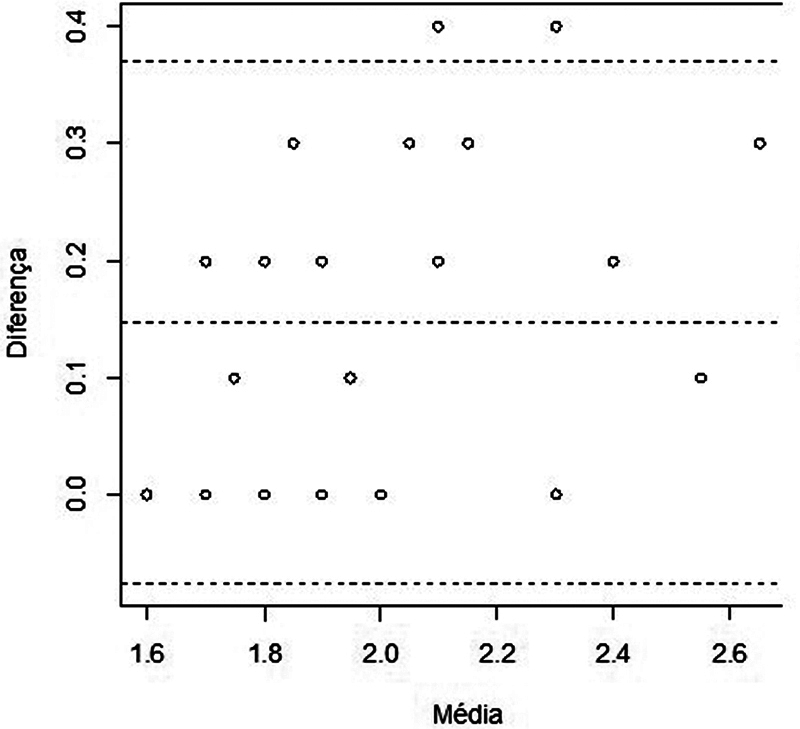
Scatter plot with limits of agreement between the measurements performed on the right side.

**Fig. 5:**
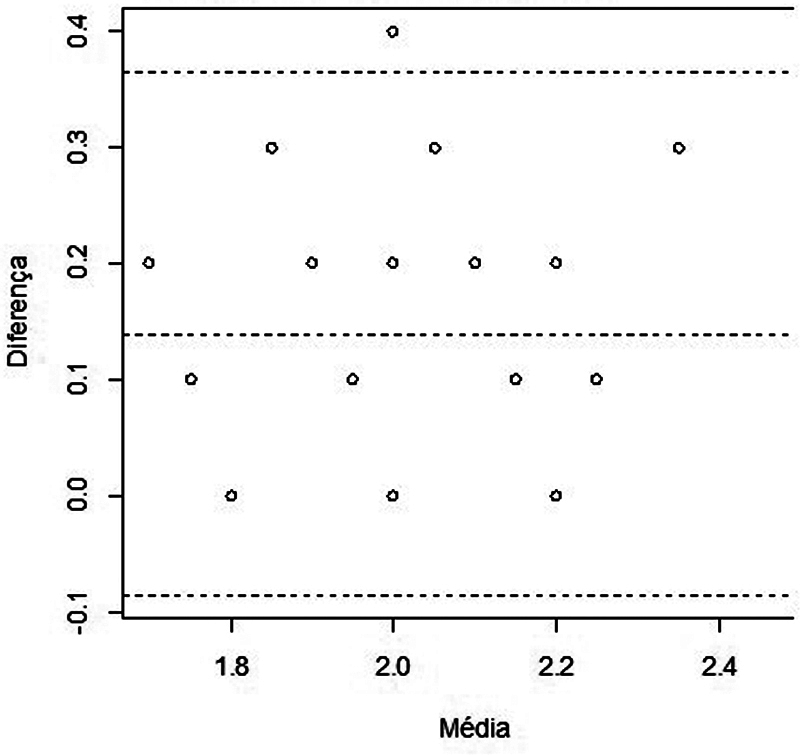
Scatter plot with limits of agreement between the measurements performed on the left side.

The Bland–Altman plots for the right and left sides are shown in Figs. 4 and 5 respectively. The horizontal central line indicates the average of the differences between the measurements and the horizontal dashed lines at the ends indicate the lower and upper concordance limits, with 95% confidence. Most of the data are within the range and the measurements are consistent.

The linear regression models were adjusted according to the differences in the measurements and their means, for the right and left sides, to assess for proportion bias ( Table 8 ).

**Table 8:** Linear regression model between differences in measurements and means

Settings	Coefficient	Standard error	*p* -value	95% confidence interval
Mean (right side)	0.12	0.07	0.112	− 0.02–0.26
Mean (left side)	− 0.06	0.10	0.555	− 0.26–0.14

The *p* -values of the adjustments were > 0.05 on both sides without proportional bias. This indicates that, for the difference in values, there are no trends. They are distributed evenly around the average of the differences.

#### Linear Regression

The linear regression models for the AB-dissection and AB-tomography measurements made by radiologist 1 in the right and left nasal cavities were adjusted ( Table 9 ).

**Table 9:** Linear regression model between measurement differences and their means

Settings	Coefficient	Standard error	*p* -value	95% confidence interval
AB-tomography (right side)	1.08	0.01	< 0.001	1.06–1.10
AB-tomography (left side)	1.07	0.01	< 0.001	1.05–1.09

For each 1-cm increase in the AB-tomography measurement, an increase of 1.08 cm on average is expected in the AB-dissection measurement (right side: 95%CI: 1.06–1.10; and left side 1.07; 95%CI: 105–1.09).

### Summary of the Findings

- The AB-dissection and AB-tomography measurements performed by two radiologists on CT scans were ≥ 1.5 cm ( Table 2 ).
- The agreement between the two radiologists was classified as excellent ( Table 4 ).
- The AB-dissection and AB-tomography measurements performed by radiologist 1 presented a strong positive correlation ( Table 6 ).
- The AB-dissection and AB-tomography measurements agree ( Figs. 4 and 5 ) and do not have proportion bias (Table 8): they are homogeneous in relation to the mean of the differences.
- For each 1-cm increase in the AB-tomography measurement, an increase of 1.08 cm on average is expected in the AB-dissection measurement ( Table 9 ) (right side: 95%CI: 1.06–1.10; and left side 1.07; 95%CI: 105–1.09).

## Discussion

The anatomical variation between the paranasal sinuses is high. 14 Anatomical variations and noble structures close to the sites of surgical access increase the chances of surgical complications. 15 These complications can cause sequelae or death of the patient. 16 17 Further comparisons of the findings of the present research findings with those of similar ones in the literature was not possible due to the absence of studies on anatomical measurements or distances.

The consistency of the AB measurements evaluated in the dissection and tomography analyses confirms the hypothesis of a regular distance between the posterior wall of the maxillary sinus and the skull base observed in surgical practice and in two studies performed on cadavers 12 and CT scans, however with a difference of 0.5 cm between them. The new research with the same measurements on CT scans and on cadavers showed that they were > 1.5 cm in all cases evaluated.

Computed tomography scans are used in the surgical practice to “map” the anatomy of the paranasal sinuses to define the surgical approach. 14 18 This exam helps with the diagnosis, the decision of the surgical treatment, and the surgeon can identify the anatomical changes and the regions with the highest risk of iatrogenic outcomes. 9

The AB-tomography measurement could be performed routinely by the radiologist by configuring the MPR parameters. The measurement could be performed in preoperative CT scans of the paranasal sinuses and used as reference by the surgeon for access the posterior sinuses for endoscopic surgery, since a strong positive correlation between this measurement on CT scans and dissections was found. The measurements obtained on CT scan are slightly shorter than on the dissections. Computed tomography scans measurements are performed with a millimeter ruler on a computer screen, and the dissection ones with a scalpel with a ruler and through observation by the researcher. The measures are analogous and positively correlated.

The AB-tomography measurement enables the surgeon to infer the safe distance to the anterior skull base, more specifically to point B. This enables access to the posterior sinuses through the identification of the posterior wall of the maxillary sinus, optimizing the sinus surgery with low complications.

The similar results of the study performed on tomography and on cadavers 12 strengthen the theory that the distance between the posterior wall of the maxillary sinus and the Δ90° at the skull base is generally > 1.5 cm. This measurement can be performed routinely on CT scans as a reference during surgeries with surgical steps established in the literature. The measurement is an additional component to be used during surgeries.

## Conclusion

The present research aimed to identify a standard distance in a region with large anatomical variations that may be used even to identify an individual. The distance between two anatomical points was assessed and the measurement was found to be constant in the cases analyzed: > 1.5 cm.

The confirmation of the regularity of the AB measure and the strong positive correlation indicate the existence of a safe anatomical reference that can be measured in the main image exam used in the preoperative period (CT scans) and in surgeries, being another safety component in paranasal sinus endoscopic surgery.
